# Evolutionary motor biases and cognition in children with and without autism

**DOI:** 10.1038/s41598-020-74224-4

**Published:** 2020-10-15

**Authors:** Gillian S. Forrester, Rachael Davis, Gianluca Malatesta, Brenda K. Todd

**Affiliations:** 1 Centre for Brain and Cognitive Development, Department of Psychological Sciences, Birkbeck, University of London, Malet Street, London, W1CE 7HX UK; 2 Department of Psychiatry, University of Edinburgh, Edinburgh, EH10 5HF UK; 3 Department of Psychological Sciences, Health and Territory, University “G. D’Annunzio” of Chieti-Pescara, Chieti, Italy; 4 Psychology Department, City, University of London, London, UK

**Keywords:** Human behaviour, Evolutionary theory, Cognitive neuroscience

## Abstract

Evolution has endowed vertebrates with a divided brain that allows for processing of critical survival behaviours in parallel. Most humans possess a *standard* functional brain organisation for these ancient sensory-motor behaviours, favouring the right hemisphere for fight-or-flight processes and the left hemisphere for performing structured motor sequences. However, a significant minority of the population possess an organisational phenotype that represents crowding of function in one hemisphere, or a reversal of the standard functional organisation. Using behavioural biases as a proxy for brain organisation, results indicate that reversed brain organisation phenotype increases in populations with autism and is associated with weaker cognitive abilities. Moreover, this study revealed that left-handedness, alone, is not associated with decreased cognitive ability or autism. Rather, left-handedness acts as a marker for decreased cognitive performance when paired with the reversed brain phenotype. The results contribute to comparative research suggesting that modern human abilities are supported by evolutionarily old, lateralised sensory-motor processes. Systematic, longitudinal investigations, capturing genetic measures and brain correlates, are essential to reveal how cognition emerges from these foundational processes. Importantly, strength and direction of biases can act as early markers of brain organisation and cognitive development, leading to promising, novel practices for diagnoses and interventions.

## Introduction

### The divided vertebrate brain

Although the causal origins are yet to be determined, comparative investigations across species suggest that the majority of vertebrate populations, including humans, inherited a ‘standard’ vertebrate brain template featuring a divided brain. This template relies on cerebral lateralisation of function which favours the left hemisphere for routine motor sequences and the right hemisphere for fight-or-flight behaviours^1^. Good division of labour between the two hemispheres affords advantages to the organism including: neural efficiency, parallel processing and the reduced chance of simultaneous and incompatible responses^2,3^. For example, the organism has the basic survival abilities to eat whilst also keeping an eye out for predators^4^.

### Cognitive exaptations through evolution

Evolutionary theory suggests that, over time, these hemisphere dominances provided a platform and the critical processing to support cognitive ‘exaptations’, which now represent modern human social and communication capabilities. Exaptation in this context refers to the co-option or shift in function of a trait during evolution^5,6^. Moreover, as a result of the contralateral control of motor-sensory processes, the study of behavioural biases provides a reliable, albeit indirect measure, of the organisation of brain function^7^ and offers a valuable opportunity to explore the causal links between brain organization, behaviour and cognitive function^8^. For example, humans recognise the identity and emotions of faces with a right hemisphere bias [for a review, see^9^], suggesting that this ability benefits from the right hemisphere’s foundational dominance for spotting danger and threat in the environment^10^. Conversely, human population-level right-handedness for tool-use and speech^11^ is supported by the left hemisphere’s dominance for producing structured and routine motor sequences^12–14^.

Social-emotional processes are fundamental and critical components of modern human behaviour. Across cultures, humans demonstrate a population-level left visual field bias and advantage for identifying faces and their emotions^15,16^. Social-emotional behavioural biases also extend to social activities such as, hugging, kissing, nurturing infants^17^ and even by proxy when cradling dolls. These behaviours favour the left side of visual space, the left arm, left cheek or side of the body—demonstrating a right hemisphere bias for producing and perceiving social stimuli. It is present across cultures [for a review, see^10^] and is generally observable in young children without significant influence of age, experience or sex. A similar pattern of behaviour is also found across an extensive range of animal species^18,19^ and in all great apes, suggesting an evolutionary old origin [for a review, see^20^]. Taken together, the evidence supports the hypothesis that modern human behaviours exhibit population-level lateral biases that extend from evolutionarily old cerebral lateralisation of function^10^.

Language processes are also fundamental and critical components of modern human behaviour. Across cultures, humans demonstrate a population-level left hemisphere anatomical and functional bias for producing and comprehending language, regardless of modality (e.g. speech, hand signs), suggesting that Broca’s region did not emerge as a language specific area in humans, but that it, and the analogous monkey brain neural region (F5), operate as a supramodal processor for routine and structured motor action sequences conducted by the hands and mouth^21,22^. The left hemisphere bias for structured motor sequences also extends to tool-using behaviours resulting in a largely right-handed population across cultures^23^. All great apes possess analogous language regions that are larger in the left hemisphere^24,25^ and also demonstrate population-level right handedness for tool use [for a review, see^26^]. A similar pattern of right-side behavioural biases is found across an extensive range of animal species for routine and structured motor sequences [for reviews, see^27–29^]. Moreover, in humans, hand biases are established at least by school age^30^, and some evidence suggests that thumb-sucking behaviour in utero is significantly associated (80%) with subsequent evaluation of hand dominance at school age^31^. Again, these findings support the hypothesis that population-level lateral biases in modern humans extend from evolutionarily old cerebral lateralisation of function and are observable early in development^10^.

### Cognitive exaptations through development

Comparative investigations of vertebrate species suggest that good division of labour across the hemispheres is associated with strong fitness and the survival of the organism^3^. Although not studied within an evolutionary framework, psychological investigations agree that the presence of cerebral lateralisation is a sign of healthy brain development^32^ and have drawn similar patterns of associations between the direction of behavioural biases and cognitive ability in humans, for example with hand dominance^33,34^. If we now entertain the evolutionary theory that foundational motor-sensory delineation across the two hemispheres lays the platform for higher cognitive functions, behavioural biases can provide an informative approach to investigate the associations between brain organisation, function and cognitive development.

One investigation that has taken this Evo-Devo approach considered the impact of brain biases on cognition using behavioural biases as an indirect measure of brain organisation. This study employed a cradling paradigm in typically developing young children^8^. Children aged four and five years-old demonstrated a population-level bias to hold a baby doll on the left (65%). Additionally, a proto-face pillow, adorned with three dots in an inverted triangle orientation surrounded by an oval boundary, elicited a left holding bias (67%), while the identical pillow without a face did not. The holding results are consistent with adult findings and suggest that the salience of the most basic face configuration^35^ is sufficient to elicit a left cradling bias in children. The results demonstrated that this behavioural bias was not only observable in young typically developing children, it was also associated with higher survey scores measuring facets of social and communication abilities (evaluated by teachers) compared with typically developing children who cradled the doll on the right. The divergence of the social scores may reflect the differences in functional brain and prompts the need for further research to understand the role of cerebral lateralisation and social cognitive development.

### Disruption to the divided brain

Deviation from the standard behavioural biases have been reported in a variety of studies but lack a systematic approach [for a review, see^36^]. Nevertheless, weakened, absent or reversed behavioural biases tend to be linked with decreased cognitive function. For example, the frequency of non-right-handedness (left-handedness and ambidexterity) rises in populations of individuals with developmental disorders, like autism^37^ and mental health conditions (e.g. schizophrenia)^38^. Moreover, historical cultural and scientific literature emanates a negative association with left-handedness even though the causal origins are unclear^39^. In fact, in addition to the disruption to typical social development, a decrease or reversal in the population-level brain and behavioural biases is a commonly identified characteristic in individuals diagnosed with autism. For example, atypical lateralisation of motor circuit functional connectivity and a rightward shift of motor circuitry has been found in fMRI studies of adults with autism^40^. This disruption of the typical brain lateralised organisation of function also extends to social processing. For example, individuals diagnosed with autism demonstrate face processing deficits associated with diminished activation of the right fusiform gyrus [for a review, see^41^] and the absence of a left visual field bias viewing face stimuli^42^.

Although motor-sensory development and its influences have not always been investigated in concert with cognitive development, there is considerable evidence that when there is a disruption to the typical development of early motor-sensory processing capabilities there will be cascading impact on the development of higher cognitive functions^43^. It now seems evident that part of the early typical development of motor-sensory processes involves lateralisation—and that *disruption* can occur in the form of a decrease or reversal in the delineation of foundational motor-sensory dominances across the two hemispheres^10,44^. However, the way in which a disruption to foundational brain biases influences the unfolding of cognitive ability is only just emerging as an area of interest for scientific study.

A recently published article has used eye tracking to evaluate the gaze side biases to face stimuli in infants at high and low risk for autism^45^. This study benefited from secondary data analysis of infants at 6 and 14 months of age from the prospective longitudinal British Autism Sibling Infant Study (BASIS) who at age 3 were established as developing (i) typically, (ii) atypically or (iii) had received a diagnosis for autism. The study reported that only infants who went on to receive an autism diagnosis did not exhibit a bias for face stimuli on the left at both timepoints. In fact, at 6 months the children with autism demonstrated a preference for stimuli on the right and were slower than their typically developing counterparts to look at faces on the left, however this group difference disappeared by 14 months. Moreover, the associations between lateral looking behaviour at 6 months and language and motor ability at 14 months were identified in the expected direction. Two points should be gleaned from this investigation. First infants who eventually receive an autism diagnosis at age 3 exhibit differences in gaze behaviour early during infancy. Second, there may be windows of opportunity to detect risk and provide interventions, during which cerebral lateralisation of function is developing.

### Directional motor phenotypes

There have been recent discussions in the review literature suggesting that a *standard* functional brain organisation template is expressed by the majority of the population and is associated with typical cognition, while disruption to the standard template ‘results in crowding’ or reversal of the standard template. Although crowding does not appear to have significant cognitive implications, reversal is associated with decreased cognitive ability^36,46^. This is consistent with the comparative literature but lacks a narrative of causal origin. Moreover, it remains a significant issue that sensory-motor (e.g. praxis) and higher cognitive functions (e.g. language) are considered independent processes. The Evo-Devo framework considers the evolutionary basis of human cognition and how that foundational motor-sensory processes still play a significant role in the cognitive development of modern human infants. As such, one would expect that higher cognitive functions like language and social-emotional processing would build upon the foundational support of the ancient lateralised vertebrate motor-sensory processes—e.g. left hemisphere motor sequencing dominance and right hemisphere fight-or-flight processes—for the most efficient cognitive outcomes.

A better understanding of the associations between behavioural biases, brain organisation/function and cognitive ability during childhood is important for identifying and tracking behavioural phenotypes to allow us to make predictions about developmental trajectories across both typical and atypical populations. This study extends the work of Forrester and colleagues^8^ to a population of children with autism. Using behavioural biases as a proxy for brain organisation, we evaluated the associations between behavioural biases and cognitive ability and compared the results of the children with autism with the typically developing (TD) population from Forrester and colleagues^8^. Additionally, we pooled data from children with and without autism to see if an analysis by directional motor phenotypes (standard, crowded, reversed^36^) could predict diagnosis. If standard and non-standard brain organisation can be systematically mapped during development and becomes a robust marker of cognitive development, it would not only add evidence to suggest that modern human abilities are dependent on evolutionarily old sensory-motor abilities, it may also lead to promising, novel practices for diagnoses and interventions. This is the first step towards such an endeavour.

## Methods

### Participants

Ninety-eight children with a diagnosis of autism (11 girls) attending one of four UK Special Educational Needs (SEN) schools participated in this study (mean age = 98 months, SD = 44.13 months, range 48–228 months). The population was proportionately male-biased, consistent with UK diagnostic statistics^47^ however, we did not expect sex differences in experimental findings. Participating schools were based in: London (High Barnet, Tower Hamlets), Surrey and Bath. One girl and nine boys declared themselves left-handed based on asking ‘what is your favourite hand for writing?’. Unlike the TD children whose hand declaration statistically matched their behavioural performance in a fine motor task (see pegboard task below), the ASD group’s self-report hand dominance was statistically different from their fine motor task hand dominance. All analyses employed the fine motor task for hand dominance analyses.

All methods were performed in accordance with the relevant guidelines and regulations of the 1964 Declaration of Helsinki. Ethical approval for the current study was authorised by the Department of Psychological Sciences Ethics Committee at Birkbeck, University of London. Parents and/or legal guardians gave informed consent for child participants. Testing procedure followed the protocol from Forrester and colleagues^8^. Full details of the tasks can be found in the original manuscript. All participants participated in the following battery of tasks:

### Pegboard task

A 10 × 10 plastic pegboard task was used to assess hand dominance for fine motor control and general praxis. Children were encouraged to place as many red pegs as they could on the outline of a red square consisting of 20 holes in 90 s. The researcher scored the number of pegs placed with the left hand and the right hand. A laterality index score (LIS) was calculated for each participant using data from the pegboard task. LIS were calculated using the formula [LIS = (R − L)/(R + L)], with R and L corresponding to the frequency of events resulting in scores ranging between − 1.0 and + 1.0 where greater positive values reflect an increasing right hand preference and greater negative values represent an increasing left hand preference. Participants were classified as right-handed for scores ≥ 0.2 and left-handed for scores ≤ − 0.2. Participants with scores between ± 0.2 were classified as ambi-preferent.

### Cradling task

This task was conducted to assess whether children exhibited a side bias for cradling social and non-social stimuli in one arm or the other—giving the left or right visual field an advantage respectively for viewing stimuli. The cradling task was comprised of three trials that involved cradling a pillow stimulus followed by a human infant and an ape infant doll, counterbalanced in presentation across participants. Each child began with either the proto-face pillow or the no-face pillow. The pillow stimuli trial was always presented first so that the cradling trials involving the dolls did not confound the pillow holding with a sense of animacy. The pillow stimuli (face or no-face) acted as a between-participant contrast, whereas the type of stimulus (pillow, human infant doll, primate infant doll) acted as a within-participant contrast. A symmetrical cradling gesture demonstrated by the researcher^48^ accompanied the relevant cradling instruction to the child who was presented with the stimulus upright and positioned towards their midline. The side where the child chose to place the head or top of the stimulus determined the ‘left’ or ‘right’ coding of the stimulus.

### Socio-communication survey

The socio-communication survey was devised to determine the social and communication abilities of each child (see Table 5^8^). The survey was comprised of 14 questions, 7 questions relating to social abilities and 7 questions relating to communication abilities. The key teacher or teaching assistant for each child completed the survey and scored each participant using a 5-point Likert scale ranging from strongly agree to strongly disagree. Categorical selections were transcribed into scores of 1–5 where high scores equated to stronger ability levels. Both the social and communication items were developed to reflect milestones for typically developing five-year olds.

### Phenotype grouping

Participants were categorised by directional motor phenotype using side biases from the individual’s pegboard laterality index score and holding side of the human infant doll. To identify cerebral lateralisation for praxis/fine motor ability bias by proxy, participants were identified as left or right-handed based on their pegboard laterality index score (L < − 0.2 > ambi-preferent < 0.2 < R). To identify cerebral lateralisation social-emotional/face processing bias by proxy, participants were classified as left or right biased cradlers based on their holding side of the human infant doll. Based on these scores, participants were categorised by directional motor phenotype: (1) standard brain function template (Left Cradle-Right Handed), crowded brain template (Left Cradle-Left Handed, Right Cradle-Right Handed) and reversed brain template (Right Cradle-Left Handed).

Behavioural biases for fine motor/praxis and social/face processing behaviours allowed for the classification of four directional motor phenotype groups^36^. All statistical analyses were conducted using SPSS (Version 26). Alpha was set at 0.05 and all test were two-tailed.

## Results

### Hand dominance

Based on performance of the pegboard task, the ASD group was statistically right-handed, but the mean laterality score (0.250 ± 0.652) had greater variability (Levene’s Test of Equality of Variance, *P* = 0.001) and trended less right-handed compared with the typically developing (TD) group (0.473 ± 0.491). Equality of variance was not met (*U* = 2734, *P* = 0.083, *r* = 0.02).

### Cradling bias

Unlike the TD group who held significantly left for the proto-face pillow and infant human doll, there was no lateral bias for the ASD group for holding any of the cradling stimuli. Moreover, a group by holding side interaction demonstrated that the TD group held the infant human doll more left than right, whereas the ASD group held the doll more right than left [*χ*^2^(2, N = 148) = 5.42, *P* = 0.020, *r* = 0.191], see Table 1.

**Table 1:** ASD and TD group laterality measures by task.

	N	Left	Right	z-score	*P* value	LI score
**ASD group**						
Pegboard task	97	634	1022	9.51	< .000	.25
No-face pillow	38	17	21	.487	.522	.11
Proto-face pillow	41	23	18	.625	.533	− .12
Infant human doll	81	34	47	1.33	.181	.16
Infant primate doll	75	34	41	.692	.489	.09
**TD group**^**a**^						
Pegboard task	98	312	858	15.93	< .000	.47
No-face pillow	44	19	25	.754	.451	.14
Proto-face pillow	37	27	10	2.63	.0076	− .46
Infant human doll	80	52	28	2.57	.0097	− .30
Infant primate doll	74	25	49	2.67	.0071	.32

^a^TD group results are taken from Forrester and colleagues.^8^.

### Socio-communication ability scores

The TD group scored higher on all social and communication ability questions compared with the ASD Group: Communication scores (*U* = 548, *P* < 0.000, *r* = 0.499), Social scores (*U* = 454.5, *P* < 0.000, *r* = 0.534). Variation in population scores were greater in the ASD compared with the TD group, see Table 2.

**Table 2:** Mean cognitive scores by group (TD data from Forrester et al.^8^).

	N	Mean score	SD	Min	Max	SE
**ASD**						
Social survey	98	20.08	6.30	0	35	.636
Communication survey	98	16.24	8.03	0	35	.811
**Typically developing**						
Social survey	66	29.84	2.83	0	35	.348
Communication survey	66	29.92	4.29	0	35	.529

Unlike with the TD group for the ASD group, social survey scores were not associated with the holding side of the infant human doll or any cradling stimuli. However, communication ability scores for left handed (13.15 ± 7.54) were weaker than right-handed children (17.44 ± 8.27) where hand classification was determined by pegboard laterality index scores (L < − 0.2 > M < 0.2 < R) (*t*_76_ = − 2.221; *P* = 0.029, *d* = 0.54, 95% CI − 8.13, − 0.44).

### Directional motor phenotypes

**Figure 1:**
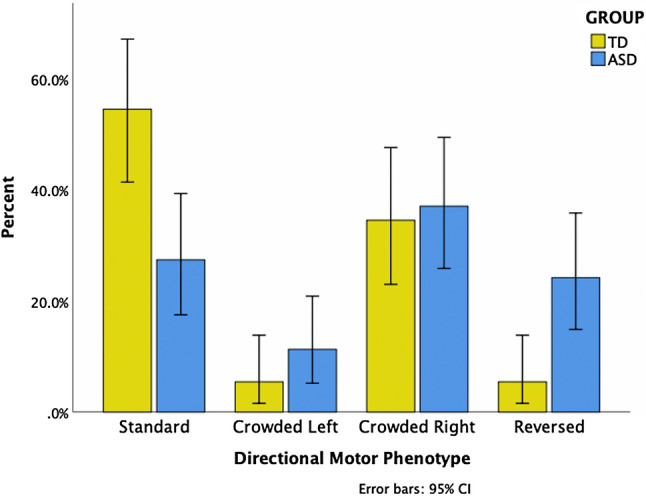
Frequency distribution of directional motor phenotypes presented as cumulative percentages by group with 95% confidence intervals.

There was a significant phenotype by group interaction [*χ*^2^(2, N = 117) = 13.05, *P* = 0.004, *r* = 0.336] see Fig. 1. Shifting the threshold of the hand dominance scores to higher (+/−0.4) or lower (+/−0.0) to decrease or increase the number of cases included in phenotyping did not change the significance of the effect of phenotype by group interaction.

Post hoc tests revealed a significant interaction of standard and reversed phenotypes by group [*χ*^2^(1, N = 65) = 11.58, *P* = 0.001, *r* = 0.422], indicating that the TD group expressed the standard phenotype more frequently than the reversed phenotype, whereas the ASD group expressed standard and reversed phenotypes with equal frequency. There was also a weaker but significant interaction between the crowded right and reversed phenotypes by group [*χ*^2^(1, N = 60) = 4.43, *P* = 0.035, *r* = 0.272], indicating that the TD group elicited significantly fewer instances of reversed phenotype compared with crowded right phenotype, whereas the ASD group expressed no difference in frequency of the two phenotypes.

One-way ANOVAs indicated that neither the ASD group nor the TD group demonstrated a significant difference in cognitive ability scores across the different phenotypes: ASD group: survey scores for communication ability [*F*(3, 58) = 2.47, *P* = 0.071] and for social ability [*F*(3, 58) = 1.81, *P* = 0.155]; TD group: survey scores for communication ability [*F*(3, 50) = 1.03, *P* = 0.384] and for social ability [*F*(3, 50) = 1.10, *P* = 0.358], see Table 3.

**Table 3:** Phenotype and survey scores by group.

Cradling bias-hand class	Brain organisation phenotype	TD Social survey N, mean, SE	ASD Social survey N, mean, SE	TD Comm survey N, mean, SE	ASD Comm survey N, mean, SE
Left–right	Standard	30, 30.21, .556	17, 21.71, 1.35	30, 30.33, .695	17, 19.06, 2.05
Left–left	Crowded left	2, 29.50, 4.50	7, 22.00, 1.89	2, 29.50, 2.5	7, 16.86, 3.31
Right–right	Crowded right	19, 28.89, .587	23, 22.35, 1.18	19, 28.37, 1.19	23, 19.04, 1.58
Right–left	Reversed	3, 28.00, .000	15, 17.93, 1.91	3, 31.67, .882	15, 12.53, 1.93

### Pooled group analyses

When disregarding diagnoses, hand classification (derived by pegboard laterality index scores: L < − 0.2 > M < 0.2 < R) became a strong predictor of cognitive ability. Left-handers demonstrated weaker social ability scores (20.23 ± 7.26) than right-handers (25.22 ± 6.47) (*t*_130_ = − 3.65, *P* < 0.000, *d* = 0.68, 95% CI − 7.70, − 2.29). Left-handers (16.00 ± 9.57) also demonstrated weaker communication ability scores compared with right-handers (23.33 ± 9.03) (*t*_130_ = − 3.90, *P* < 0.000, *d* = 0.79, 95% CI − 11.05, − 3.61).

This finding did not appear to be driven by an increase in left hand frequency in the pooled dataset. Hand laterality index score (derived from pegboard task) was also positively correlated with social survey scores (*r* = 0.223, *P* = 0.004) and communication survey scores (*r* = 0.219, *P* = 0.005).

The variability of cognitive and laterality scores was higher in the ASD group compared with the TD group. Pooling data allowed for enough power for ambi-preferent individuals (− 0.2 > ambi-preferent < 0.2) to be analysed as a group. The ambi-preferent group (22.45 ± 9.36) was not significantly different from the right-handed group but was significantly stronger compared with the left-handed group (16.00 ± 9.57) for communication ability scores (*t*_60_ = 2.68, *P* < 0.009, *d* = 0.681, 95% CI − 11.26, − 1.64) and also trended in the same fashion for social ability scores (23.81 ± 9.36) compared with the left-handed group (20.23 ± 7.26) (*t*_60_ = − 1.89, *P* = 0.064, *d* = 0.48, 95% CI − 7.37, 0.21), see Table 3.

A one-way ANOVA for pooled group data revealed a significant effect of phenotype by survey scores for communication ability [*F*(3, 112) = 7.25, *P* < 0.000, *d* = 0.41] and for social ability [*F*(3, 112) = 6.87, *P* < 0.000, *d* = 0.36] see Table 3. Effect sizes calculated using group mean information (sample size, mean, variance), see Table 4.

**Table 4:** Phenotype by survey scores (group data pooled).

Cradling bias-hand class	Brain organisation phenotype	N	Social survey Mean, SE	Comm survey Mean, SE	Total survey Mean, SE
Left–right	Standard	47	27.13, .847	26.26, 1.17	53.38, 1.97
Left–left	Crowded	9	23.67, 1.97	19.67, 3.16	43.33, 4.89
Right–right	Crowded	42	25.31, .857	23.26, 1.24	48.57, 1.95
Right–left	Reversed	18	19.61, 1.82	15.72, 2.36	35.33, 4.02

**Figure 2:**
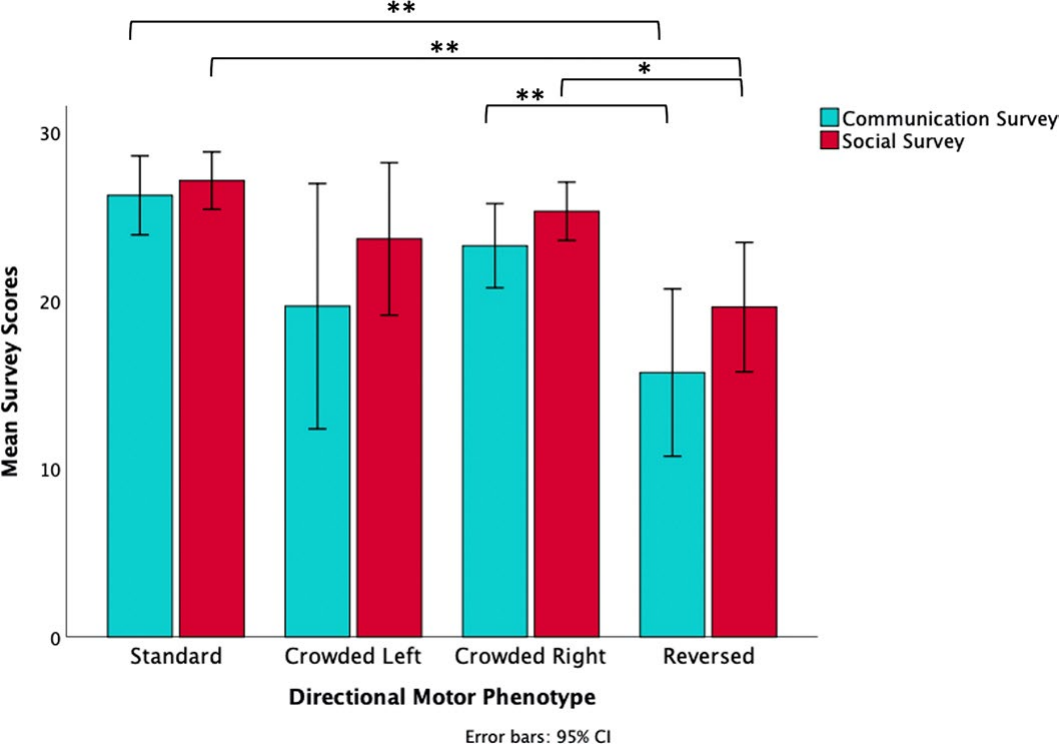
Mean scores of social and communication surveys by directional motor phenotype with 95% confidence intervals.

Post hoc tests (Tukey HSD) revealed significant differences between standard and reversed phenotypes for both the communication ability scores (*P* < 0.000) and social ability scores (*P* < 0.000). Significant differences were also revealed between crowded right and reversed phenotypes for both the social ability scores (*P* = 0.006) and the communication scores (*P* = 0.011), see Fig. 2. Shifting the threshold of the hand dominance scores to higher (+/−0.4) or lower (+/−0.0) to decrease or increase the number cases included in phenotyping did not alter the effect of phenotype on cognitive scores.

Independent samples t-tests indicated no sex differences within groups for any of the dependent measures.

## Discussion

This investigation sought to extend the literature regarding the associations between behavioural biases and cognitive ability.

### Population comparisons

Results suggested that, as a population, children with autism exhibit significantly weaker hand dominance and a higher incidence of non-right-handedness. This is consistent with previous findings^33,34^ and can be interpreted as reflecting decreased or reversed cerebral lateralisation of function for motor sequencing/praxis. While there was no significant group difference in the number of pegs placed in the pegboard task, it should be noted that the age range of the ASD group was much greater than that of the TD group which may have influenced this result.

Children with autism also demonstrated a lack of bias for cradling a human infant doll or a proto-face pillow, both of which elicited a significant bias in the TD group in Forrester and colleagues’ investigation^8^. This result is also consistent with previous investigations of social-emotional processing, including cradling, where individuals with autism possess weakened, absent or reversed side bias^48^ and is thought to reflect weakened, absent or reversed cerebral lateralisation of social-emotional function.

Children with autism, predictably, scored lower on both the social and communication surveys. However, in this group the cradling side was not associated with social survey ability scores—as was found with the TD children. This may because the cradling measure only features side (left, right) and not strength of bias because it is based on a single trial. Instead we see a link between hand dominance and cognitive scores, which was not observed in the TD group. Both the social and communication ability scores were higher in right-handed participants and the strength of the handedness was positively associated with the survey scores. It is possible that this pattern did not reveal itself in the TD group because those children, on the whole, were strongly right-handed with little variability within the group. However, it should not be concluded that right-handedness is a marker of typical cognitive development and left-handedness is associated with atypical cognitive development. Firstly, this result comes from within the atypical group and was not observed in relation to left-handedness in the TD group. Second, there is additional information to be gleaned from the parcellation of individuals into phenotype groups.

### Directional motor phenotypes

Pegboard and cradling results indicated individual motor-sensory biases and allowed for the categorisation of participants by directional motor phenotypes. We considered the behavioural biases to reflect the ancient dominances of the contralateral hemispheres. Based on evolutionary theory of the survival of the ‘divided brain’, we expected the standard phenotype, which consists of a left hemisphere dominance for a range of behaviours associated with motor production and a right hemisphere dominance for a range of behaviours associated with social-emotional processing, to occur most frequently^1^. We also hypothesised that the phenotypes would impact differently upon behaviour and cognition. With evidence that foundational motor-sensory lateralisation is associated with physical fitness of the organism, we hypothesised that this advantage would be extrapolated to ability level in higher cognitive functions supported by foundational motor-sensory processes. Therefore, we expected standard and reversed phenotypes to outperform crowded phenotypes for cognitive ability.

Recent research has revealed the crowded phenotype, where both motor and social abilities are dominant to the same side (left or right), to be the second most frequent functional brain organisation with the reversed template (where motor is right hemisphere dominant and social processing is left hemisphere dominant) to be the least represented^36,46^. This may seem contrary to comparative research and evolutionary theory, which would predict that the standard and reversed phenotypes would be most advantageous for efficient behaviour because each hemisphere is dominant for a primary survival function, regardless of side. Although population-level directional asymmetries may have arisen as an ‘evolutionarily stable strategy’ of behavioural alignment within a population^49,50^, reversed phenotypes may have emerged as an evolutionarily mechanism of frequency-dependent selection such that certain heritable variants can benefit only if they occur less commonly^51,52^. In this scenario, the lower incidence of non-standard phenotypes in directional asymmetries might have represented an evolutionarily advantage by making individuals’ behaviours less predictable^53,54^. It is also possible that modern humans no longer rely on the same ancient behaviours for survival and therefore crowded phenotypes are preserved within the population. However, it is also important to note that the majority of the human studies have not focused on multiple behavioural biases, nor have they differentiated between motor-sensory biases and higher cognitive function biases. Therefore, a clear picture of the frequency and distribution of directional motor phenotypes across the population has yet to be revealed. Nor do we have any clarity on how the development of higher cognitive functions unfold upon early motor-sensory brain biases.

This frequency of phenotypes described by Vingerhoets^36^ was true for the TD group, however crowding to the right was more common than crowding to the left. The population pattern for directional motor phenotypes in the ASD group was significantly different. Children with autism demonstrated equal propensity to possess crowded right, standard or reversed phenotypes. Children with autism were least likely to possess a left crowded functional brain organisation. Analyses of motor phenotype within groups indicated no significant differences in social and communication ability based on brain organisation. The significant difference in the representation of the motor phenotypes across groups begs the question of biological causal origins and further studies could address these questions directly by conducting genetic studies that recruit participants from each of the four phenotypes, with special attention to the case of reversed phenotype.

The next set of analyses involved the pooling of data from the two groups into a single dataset for analyses by directional motor phenotype. The rationale for such an approach is that the TD group data has little variability, as the population tends to be strongly right-handed and also perform at the top of the scale on social and cognitive ability milestones, creating ceiling effects that can mask latent patterns in the data. Pooling the data from the TD and ASD groups amplified the association between hand classification and strength of hand dominance with cognitive ability scores. However, it also revealed that it is not left-handedness that was the influencing factor, but rather left-handedness as a marker of reversed brain organisation phenotype. Therefore, left-handedness alone is not associated with decreased cognitive ability or autism, nor when it is associated with the crowded left brain phenotype. This is an important point because there is a long history of cultural bias drawing associations between left-handedness and cognitive and psychiatric disorders^39,55^. The current study suggests that that left-handedness can manifest from two different patterns of cerebral lateralisation: (1) crowded left, which poses no significant cognitive differences to the standard or crowded right brain organisation phenotypes or (2) reversed, which may be a marker of disruption to typical brain organisation and consequently cognitive ability. Hand dominance is just one indirect marker of functional brain organisation.

The results from this study suggest that individuals with autism appear to have a higher frequency of reversed functional brain lateralisation than typically developing children. However, this pattern exists also within the typically developing population and when data are pooled, it demonstrates that this phenotype is associated with lower social and communication ability compared with standard and crowded functional brain templates. As such, behavioural biases as indirect makers of brain organisation early in development may provide a useful early marker of risk for developmental conditions like autism.

### Development within an evolutionary framework (evo-devo)

It seems, however, that we are still missing a piece of the puzzle. Current and past studies lump all types of behavioural biases into experimental paradigms without consideration of the evolutionary origins of our brain biases^36^. If we consider directional motor phenotypes as an indicator of cerebral lateralisation of function, there needs to be a causal origin theory underpinning why the majority of the population possess same side dominances for a large range of seemingly disparate behaviours. Comparative studies and evolutionary theory can provide this narrative.

Moreover, a systematic approach to investigations that distinguishes foundational motor-sensory behaviour from higher cognitive functions that rely upon these foundational processes is required. The standard brain template should be drawn from the robust patterns reported in comparative investigations—where population biases of the typical vertebrate brain predict a left hemisphere dominant for routine and structured motor sequencing (e.g. feeding) and a right hemisphere dominant for recognising danger in the environment (fight/flight). What we should be asking is: (1) Are evolutionarily retained motor-sensory functional brain biases dependent, at least in part, on genetic factors? (2) During development are higher cognitive abilities taking advantage of these ancient dominances? (3) If so, how and when? and (4) What happens to higher cognitive functions when the motor-sensory biases are disrupted?

Although not framed within an evolutionary lens, the literature demonstrates that the majority of the population possesses a standard motor direction phenotype—with face processing and social-emotional abilities dominant within the right hemisphere, supported by the ancient fight-or-flight mechanism. Additionally, we see that motor sequencing and language abilities aligned in the left hemisphere because language is a higher cognitive exapted from hierarchical sequencing abilities of the left hemisphere. Therefore, future investigative approaches should consider the individual’s foundational directional motor phenotype. The phenotype itself may not be a marker of cognitive ability—however, how the development of higher cognitive functions unfold upon this template may be of significance. Therefore, a second requirement is to determine the congruency of the higher cognitive functions with the given phenotype. Here we can hypothesise that for efficient cognitive performance, higher cognitive functions should have hemisphere dominant congruency with the foundational processes. Where there is a reversed foundational template and/or incongruency between foundational biases and higher cognitive functions we may expect domain-specific decreased cognitive ability. Without an understanding of these relationships, Hernandez and colleagues demonstrated that children with a left hemisphere motor bias but right hemisphere dominant language had inferior reading speed, accuracy and comprehension than those who had both processes converge in one hemisphere—regardless of the side^56^. It is highly probable that we have missed important relationships between behavioural biases and cognitive ability in past studies because we were not asking the pertinent questions.

If standard and non-standard functional brain organisation can be identified early in development and the unfolding of higher cognitive functions upon early sensory and motor brain biases can be systematically mapped during development, we may reveal robust markers of cognitive ability and pave new avenues of diagnostic practices and therapeutic interventions. In order to achieve these goals, we need to view humans as animals with an evolutionary history, who are dependent on ancient sensory-motor responses that lay a foundation for the development of our human-unique cognitive capabilities.

## Data Availability

The datasets generated and/or analysed during the current study are available from the corresponding author on reasonable request.

## References

[1] Rogers LJ, Vallortigara G, Andrew RJ (2013). Divided Brains: The Biology and Behaviour of Brain Asymmetries.

[2] Rogers LJ (2002). Lateralization in vertebrates: its early evolution, general pattern, and development. Adv. Stud. Behav..

[3] Vallortigara G, Rogers LJ (2005). Survival with an asymmetrical brain: advantages and disadvantages of cerebral lateralization. Behav. Brain Sci..

[4] MacNeilage PF, Rogers LJ, Vallortigara G (2009). Origins of the left and right brain. Sci. Am..

[5] Gould SJ, Vrba ES (1982). Exaptation—a missing term in the science of form. Paleobiology.

[6] Finlay BL (2007). Endless minds most beautiful. Dev. Sci..

[7] Hellige JB (1993). Unity of thought and action: varieties of interaction between the left and right cerebral hemispheres. Curr. Dir. Psychol. Sci..

[8] Forrester GS, Davis R, Mareschal D, Malatesta G, Todd B (2019). The left cradling bias: an evolutionary facilitator of social cognition?. Cortex.

[9] Demaree HA, Everhart DE, Youngstrom EA, Harrison DW (2005). Brain lateralization of emotional processing: historical roots and a future incorporating “dominance”. Behav. Cognit. Neurosci. Rev..

[10] Forrester GS, Todd B, Forrester GS (2018). Comparative approaches to lateral biases in social behaviour: a new perspective. Cerebral Lateralization and Cognition: Evolutionary and Developmental Investigations of Motor Biases. Progress in Brain Research Book Series.

[11] Forrester GS, Quaresmini C, Leavens DA, Mareschal D, Michael MSC (2013). Human handedness: an inherited evolutionary trait. Behav. Brain Res..

[12] Corballis MC (2002). From Hand to Mouth: The Origins of Language.

[13] Greenfield PM (1991). Language, tools and brain: the ontogeny and phylogeny of hierarchically organized sequential behavior. Behav. Brain Res..

[14] Higuchi S, Charminade T, Imamizu H, Kawato M (2013). Shared neural correlates for language and tool use in Broca’s area. NeuroReport.

[15] Ley RG, Bryden MP (1979). Hemispheric differences in processing emotions and faces. Brain Lang..

[16] Bourne VJ (2008). Chimeric faces, visual field bias, and reaction time bias: have we been missing a trick?. Laterality.

[17] Packheiser J (2020). Asymmetries in social touch—motor and emotional biases on lateral preferences in embracing, cradling and kissing. Laterality.

[18] Giljov A, Karenina K, Malashichev Y (2018). Facing each other: mammal mothers and infants prefer the position favouring right hemisphere processing. Biol. Lett..

[19] Salva OR, Regolin L, Mascalzoni E, Vallortigara G (2012). Cerebral and behavioural asymmetries in animal social recognition. Comp. Cogn. Behav. Rev..

[20] Hopkins WD (2004). Laterality in maternal cradling and infant positional biases: implications for the development and evolution of hand preferences in nonhuman primates. Int. J. Primatol..

[21] Pulvermüller F, Fadiga L (2010). Active perception: sensorimotor circuits as a cortical basis for language. Nat. Rev. Neurosci..

[22] Petersson KM, Folia V, Hagoort P (2012). What artificial grammar learning reveals about the neurobiology of syntax. Brain Lang..

[23] McManus IC (2002). Right Hand, Left Hand: The Origins of Asymmetry in Brains, Bodies, Atoms, and Cultures.

[24] Cantalupo C, Hopkins WD (2001). Asymmetric Broca's area in great apes. Nature.

[25] Spocter MA (2010). Wernicke's area homologue in chimpanzees (Pan troglodytes) and its relation to the appearance of modern human language. Proc. Biol. Sci..

[26] Hopkins WD, Shackelford T (2007). Hemispheric specialization in chimpanzees’ evolution of hand and brain. Evolutionary Cognitive Neuroscience.

[27] Rogers LJ, Andrew JR (2002). Comparative Vertebrate Lateralization.

[28] Versace E, Vallortigara G (2015). Forelimb preferences in human beings and other species: multiple models for testing hypotheses on lateralization. Front. Psychol..

[29] Stancher G, Sovrano VA, Vallortigara G (2018). Motor asymmetries in fishes, amphibians, and reptiles. Prog. Brain Res..

[30] Gudmundsson E (1993). Lateral preference of preschool and primary school children. Percept. Mot. Ski..

[31] Hepper PG, Wells DL, Lynch C (2005). Prenatal thumb sucking is related to postnatal handedness. Neuropsychologia.

[32] Toga AW, Thompson PM (2003). Mapping brain asymmetry. Nat. Rev. Neurosci..

[33] Rodriguez A (2010). Mixed-handedness is linked to mental health problems in children and adolescents. Pediatrics.

[34] Forrester GS, Pegler R, Thomas MSC, Mareschal D (2014). Handedness as a marker of cerebral lateralization in children with and without autism. Behav. Brain Res..

[35] Johnson MH, Dziurawiec S, Ellis H, Morton J (1991). Newborns' preferential tracking of face-like stimuli and its subsequent decline. Cognition.

[36] Vingerhoets G (2019). Phenotypes in hemispheric functional segregation? Perspectives and challenges. Phys. Life Rev..

[37] Lindell AK, Hudry K (2013). Atypicalities in cortical structure, handedness, and functional lateralisation for language in autism spectrum disorders. Neuropsychol. Rev..

[38] Ravichandran C, Shinn AK, Öngür D, Perlis RH, Cohen B (2017). Frequency of non-right-handedness in bipolar disorder and schizophrenia. Psychiatry Res..

[39] Kushner HI (2017). On the Other Hand: Left Hand, Right Brain, Mental Disorder, and History.

[40] Floris DL (2016). Atypical lateralization of motor circuit functional connectivity in children with autism is associated with motor deficits. Mol. Autism.

[41] Curby K, Willenbockel V, Tanaka J, Schultz R, Gauthier I (2010). Face processing in autism: insights from the perceptual expertise framework. Perceptual Expertise: Bridging Brain and Behaviour.

[42] Dundas E, Gastgeb H, Strauss MS (2012). Left visual field biases when infants process faces: a comparison of infants at high- and low-risk for autism spectrum disorder. J. Autism Dev. Disord..

[43] D’Souza D, Karmiloff-Smith A (2011). When modularization fails to occur: a developmental perspective. Cognit. Neuropsychol..

[44] Malatesta G (2020). Received cradling bias during the first year of life: a retrospective study on children with typical and atypical development. Front. Psychiatry.

[45] Donati G, Davis R, Forrester GS (2020). Gaze behaviour to lateral face stimuli in infants who do and do not gain an ASD diagnosis. Sci. Rep..

[46] Gerrits R, Verhelst H, Vingerhoets G (2020). Mirrored brain organization: statistical anomaly or reversal of hemispheric functional segregation bias?. PNAS.

[47] Taylor B, Jick H, MacLaughlin D (2013). Prevalence and incidence rates of autism in the UK: time trend from 2004–2010 in children aged 8 years. BMJ Open.

[48] Pileggi L-A, Malcolm-Smith S, Solms M (2015). Investigating the role of social-affective attachment processes in cradling bias: the absence of cradling bias in children with autism spectrum disorders. Laterality.

[49] Ghirlanda S, Vallortigara G (2004). The evolution of brain lateralization: A game-theoretical analysis of population structure. Proc. R. Soc. Lond. Biol..

[50] Ghirlanda S, Frasnelli E, Vallortigara G (2009). Intraspecific competition and coordination in the evolution of lateralization. Philos. Trans. R. Soc. B.

[51] Smith JM, Smith JMM (1982). Evolution and the Theory of Games.

[52] Wilson DS (1998). Adaptive individual differences within single populations. Philos. Trans. R. Soc. B.

[53] Vallortigara G (2019). Phenotypes in hemispheric functional segregation as by-products of the evolution of lateralization population structure: comment on "Phenotypes in hemispheric functional segregation? Perspectives and challenges" by Guy Vingerhoets. Phys. Life Rev..

[54] Vallortigara G, Rogers LJ (2020). A function for the bicameral mind. Cortex.

[55] Markou P, Ahtam B, Papadatou-Pastou M (2017). Elevated levels of atypical handedness in autism: meta-analyses. Neuropsychol. Rev..

[56] Hernandez S, Camacho-Rosales J, Nieto A, Barroso J (1997). Cerebral asymmetry and reading performance: effect of language lateralization and hand preference. Child Neuropsychol..

